# Stressful Experiences, Connection, and Depressive Symptoms Among Taiwanese Han and Indigenous Youth

**DOI:** 10.3389/fpsyg.2021.695751

**Published:** 2021-08-23

**Authors:** Pei-Jung Yang

**Affiliations:** Graduate Institute of Social Work, National Chengchi University, Taipei, Taiwan

**Keywords:** depression, stressful events, connection, adolescence, indigenous, person-centered approach

## Abstract

This study examined Taiwanese Han and indigenous (Tayal) youth’s experiences of stressful life events, the association between stressful experiences and depressive symptomology, and also the indirect and interactive effects of connection on the relationship between stressful experiences and depressive symptomology. Taiwanese Han (97%) is the majority group, whereas indigenous people make up 2.3% of Taiwan’s population. Taiwanese indigenous people have experienced disparities across socio-historical, educational, and economic aspects of their lives. This study included 291 eighth-grade participants (40% from the Tayal tribe, 48.8% female, and *M*_age_ = 13.44). The Han sample in this study all lived in cities, and the Tayal sample all lived in the tribal areas of the Northern Taiwan mountain regions. Person-centered (latent class analyses) and cumulative (sum of items) approaches were used to investigate family and school stressful events, respectively. Hierarchical regression analyses were conducted separately for the Han and Tayal participants to examine the role of family and school connection in relation to family and school stressors and depressive symptomology. Our results showed that stressful experiences are clearly linked to depressive symptomology and family connection was important to both Tayal and Han youth in supporting their coping with depressive symptoms. However, Tayal youth might be particularly vulnerable to family stressful events because family stressors disrupted their connection with their parents and thereby minimized the protective function of family relationships. To decrease the likely onset of depression during early adolescence, our results suggest that it is important for parents and other family members to monitor adolescents’ daily experiences of stress and provide support when needed. Furthermore, mental health interventions need to be tailored specifically for youth in specific racial, social, and economic contexts. Tayal youth mental health might benefit particularly from increasing school connection and decreasing stresses experienced in family contexts. Implications, limitations, and recommendations for future research are discussed.

## Introduction

The reporting rate of depressed mood, depressive syndromes, and depressive disorders increases substantially in adolescence. During early adolescence, there is a particular surge in the expression of sadness and unhappy or dysphoric mood (Kandel and Davies, 1982; Peterson et al., 1993). Depression is rarely a single-onset episode. It is known to recur, and the rate of recurrence increases with each additional episode (Kessler, 2002). It is therefore important to understand the etiology of adolescent depression, and try to intervene early to decrease the likelihood of any onset during adolescence.

Adolescence naturally features many physical and psychosocial changes. While these are an ongoing part of development, their extent and magnitude are pronounced in early adolescence when compared with childhood. Furthermore, adolescence is a period of heightened sensitivity to the self in relation to others. In addition to the normative developmental changes during this period, adolescents perceive any interpersonal conflicts or any threats or challenges to their relationships with peers or family more intensely (Compas and Wagner, 2017). These developmental and interpersonal changes might feel unsettling, and adolescents might experience a sense of lacking control. Without timely or appropriate support, adolescents could feel overwhelmed and perceive the situation as stressful. Early adolescence is thus characterized as a period of vulnerability to many heterogenic types of stress exposures in developmental, interpersonal, and social contexts (Compas and Wagner, 2017; Peterson et al., 2017).

Early adolescence is developmentally an important period for the investigation of depressive mood and symptoms. This study investigated stressful events experienced by 13- to 14-year-old adolescents and examined whether feeling connected to either their family or their school may mediate or moderate adolescents’ depressive mood and behavior.

Furthermore, this study included samples from the majority Han and minority indigenous populations in Taiwan. Taiwan, an island nation neighboring the southeastern coast of mainland China, has a predominant (97%) ethnic population of Han. The majority of Taiwan’s inhabitants are descendants of Han immigrants from mainland China dating from the late sixteenth century. Although indigenous people in Taiwan currently make up just 2.3% of the population, they lived in Taiwan long before the arrival of the mainland Han people. Taiwan’s indigenous people are a branch of the Austronesian people. They have their own language, rituals, and modes of living, and have experienced disparities across socio-historical, educational, and economic aspects of their lives. This study aimed to further examine racial/ethnic differences in the association between stressful experiences, connection, and depressive symptomology.

### Stressful Experiences and Depression

Depressive episodes during the onset period seem to be accounted for by stressful experiences (Stroud et al., 2008). Adolescent research therefore tends to use social-causation theory to explain the association between stressors and depressive symptoms (Clements et al., 2008). A wide range of stressful experiences and varied stressors have been investigated during adolescence. For example, (1) daily hassles are considered as proximal stress as these incidents happen frequently in an adolescent’s proximal environments (e.g., Carter et al., 2006); (2) major incidents, such as parental divorce, are considered as critical stress as these incidents impose critical impact on an adolescent’s regular routines (e.g., Wagner et al., 1988); and (3) another type of stress is characterized by feeling a loss of the sense of control, which is often connected to actual losses such as death of loved ones (e.g., Clements et al., 2008). The link between these heterogenic types of stressors and psychological symptomology has been supported by some studies (e.g., Compas, 1987; Carter et al., 2006), but the link is unclear in other research (e.g., Compas et al., 1989; Seiffge-Krenke, 2000). These inconsistent findings might be related to the interconnected nature of stressful experiences. For example, Wagner et al. (1988) found that major negative events and daily hassles were interrelated. They suggest that major negative events might disrupt daily routines pressuring individuals to readjust their schedule or shuffle resources to maintain daily functioning, thus leading to daily hassles. Therefore, stressful experiences, such as critical events and daily hassles, are intertwined and an integrative model of stress is likely to be associated more consistently with the presence of depressive affect and behavior (Wagner et al., 1988; Seiffge-Krenke, 2000).

The Specificity Principle emphasizes that specificity is inevitable in developmental processes, because momentary interaction is determined by the specific individuals in their specific contexts at specific time and place in development (Bornstein, 2017). Indeed, if two individuals experience an identical stressful event, their perception and interpretation of or response to the event would not be the same. In addition to the nature of the event, many other factors play a part in determining the extent and magnitude of stress for that specific individual at that specific time and in that specific context. For example, memories of past unpleasant experiences might instigate anticipatory stress responses (Dedovic et al., 2009), or available support in the immediate surroundings might mitigate and decrease the perceived level of stress (Grant et al., 2006). The perceived level of stress and depressive symptomology might differ by gender too. Prior research has indicated that female adolescents experience more interpersonal stress and depressive symptoms (Kandel and Davies, 1982; Liu and Alloy, 2010). These perhaps can explain why categorization of stressful events alone could not consistently predict depressive symptomology. Stressful experiences, though often described as separate heterogenic types of events, are interconnected and effect an individual’s psychological and social adjustment holistically. Therefore, stressful events should be analyzed as an integral whole rather than as discrete events.

Past adolescent research has used cumulative approaches to examine stressful events holistically. However, cumulative approaches have not yielded consistent results. The number of stressful events did not consistently link to depressive symptomology (Schilling et al., 2008). Another way to examine stressful events as a whole is through person-centered approaches (i.e., latent class analysis). Person-centered approaches allow distinct sets of co-occurring stressful events to be uncovered and may thus provide a more accurate representation of the ways stress is experienced in life (Clements et al., 2008).

Home and school are where Taiwanese adolescents spend most of their time during early adolescence. This study used cumulative and person-centered approaches in our examination of domain-specific stressful experiences. By examining stressors in their specific domains, this study hoped to provide some insights into the etiology of depressive mood and symptoms during early adolescence. Additionally, by using both cumulative and person-centered approaches, this study aimed to understand the differences in findings when numbers or sets of stressful events are used as the unit of analyses, hoping to shed light on the inconsistent findings reported between stressful experiences and adaptation outcomes.

### Connection and Depression

Connection is one of the Five Cs (Character, Competence, Confidence, Connection, and Caring) identified by the Five Cs Model of Positive Youth Development (PYD). The Five Cs Model of PYD argues that when the Five Cs are developed over time (through setting conditions and processes that specifically fit an adolescent’s strengths and developmental needs), adolescents are likely developing toward a healthy trajectory where they are less likely to develop depressive symptoms or engage in risky behavior, and moving toward becoming a contributing member in their society (Phelps et al., 2007). Research using the Five Cs Model of PYD has consistently reported negative associations between connection and depressive symptoms (Geldhof et al., 2014; Erentaitė and Raižienė, 2015; Holsen et al., 2017). Specifically, Holsen et al. (2017) reported that connection experienced within home and school contexts demonstrated moderate negative effects on depressive and anxiety symptoms.

Indeed, connection brought forth feelings of security and stability, which may serve as inner strength and external support when individuals are under stress trying to cope with daily hassles, major life events, or something unexpected. Research has found that positive relationships are most important to adolescent adaptation, independent of ethnicity or minority status (Motti-Stefanidi, 2015). Positive supportive relationships can mitigate stress (Gutowski et al., 2018), and, of all kinds of support, the quality of family support and relationships is most important (Grant et al., 2006), particularly under conditions when the incident(s) arouses high levels of stress, jeopardizing the individual’s sense of security and stability (Tsai et al., 2018).

The literature from both PYD and adolescent stress research acknowledges the importance of connection to adolescent adaptation. Grant et al. (2006) specifically call for the inclusion of both moderator and mediator research when examining the association between stressful experiences and adolescent psychopathology. According to Grant et al. (2006), the moderating effect of contextual variables (e.g., social support) was inconclusive across past child and adolescent research, but the mediating effect of family relationships has been more consistently reported.

Connection, in this study, was examined with respect to its direct, moderating, and mediating effects on depressive symptomology, in order to provide increased understanding of the complex role played by connection in situations where adolescents’ mental health outcomes are affected by stressful experiences. However, connection as a mediator in this study was being examined not in a statistical sense, but rather in what MacCorquodale and Meehl (1948) describe as a “theoretical” discussion, meaning that we suspect that connection might explain unobserved relationship between stressful experiences and depressive symptomology but we do not infer causal inference between the variables. To ensure rigorous use of language, we use “indirect effects” rather than “mediating effects” in this article.

This study also questioned whether the role of connection manifests differently across the indigenous and Han samples.

### Indigenous Population and Depression

Research indicates that racial/ethnic minorities are exposed to more stress and greater stress. When compared with majority groups, minorities have encountered multiple interlinked stressful conditions or events (Smith and Carlson, 1997; Meyer et al., 2008), which are often precipitated by their underprivileged status relating to stigmatizing racial/ethnic experiences, disadvantaged socio-economic backgrounds, or being deprived of opportunities due to their residential locations. Research on American Indian adults has identified that stressful life events, such as having a close friend or relative die in the past year or being involved in life-threatening accidents, likely led to depressive symptoms (Whitbeck et al., 2002). Around Him and DeMand (2018) have found that, within the child population of the United States, American Indian (AI) and Alaska Native (AN) children are more likely to live in poverty, observe domestic violence, live with substance abusers, lose a parents, or experience unstable living arrangements due to parental divorce. According to research on children and youths from disadvantaged groups, these adverse events increase the chances of developing depressive symptoms, substance abuse problems, or other negative psychological or health outcomes (Garćia Coll et al., 1996; DuBois et al., 2002).

Indigenous children in Taiwan experience similar socio-economic and domestic hardships to those experienced by AI/AN children in the United States. According to The Council of Taiwan Indigenous People (2019), indigenous households are more likely than the average Taiwanese household to be in the lower socio-economic range. Most indigenous parents do not undertake post-18 or higher education and are employed in labor-intensive positions in the manufacturing or construction industries. Such work environments have increased health and safety hazards, resulting in accidents (even death) being more frequently reported. This perhaps explains why the life expectancy of the Taiwan indigenous population (70.86years) is ten years less than the population average (80.69years; Taiwan Ministry of Interior, 2020a,b). Reports from both The Council of Taiwan Indigenous People (2019) and the Taiwan Ministry of Interior (2020a,b) suggest that indigenous children in Taiwan, similar to AI/AN children in the United States, may encounter multiple socio-economic stressors in their family and educational contexts. Indigenous children are likely to experience deaths of family members earlier than Han children, and they, like their parents, may find it difficult to adjust to or continue in mainstream education. All these stressors are potential sources for emotional or psychological difficulties, such as depression.

The indigenous sample in this study was from the Tayal tribe, which is the third largest of the 16 indigenous tribes in Taiwan. The Tayal people mainly reside in the Northern part of Taiwan. The Tayal adolescents in this study all lived in the Northern Taiwan mountain regions, where shortages in extracurricular activities or employment opportunities are common. Tayal parents often have to leave their tribes in search of employment in the cities. Not all parents can afford to bring their children with them; therefore, many Tayal children remain in the tribes under the care of their grandparents. Schools in the mountain regions are also not easily accessible for all tribal children. While there tends to be one elementary school per tribal community, junior high schools often require the tribal children to commute some distance. Not all Tayal children can afford the daily commute to junior high school. So, some choose to board at school or live with relatives. The school curriculum tends to revolve mainly around mainstream Han culture. Education reformists have advocated for a culturally inclusive curriculum, and indigenous activists have requested that indigenous tribal schools have more freedom to include culturally specific materials in their lesson plans (Yang and Ong, 2019).

Research on indigenous children and adolescents is scarce. This study, by examining stressful experiences as a whole (i.e., cumulative and person-centered approaches) and the complex role of connection, aimed to provide a greater racial/ethnic understanding of the risk and protective factors on adolescent depressive symptomology during early adolescence.

## Materials and Methods

### Participants and Procedure

This study included 291 eighth-grade participants (40% from the Tayal tribe, 48.8% female, and *M*_age_ = 13.44). During recruitment, several junior high schools in cities and tribal areas in the Northern part of Taiwan were contacted and five schools agreed to participate in the study (two urban schools and three tribal schools, comprising 10 eighth-grade classes). All students and their parents were informed about the purpose and procedure of the study. Students whose parents consented to their participation subsequently participated in the study. All participating students also provided assent. The study received ethical approval from the University’s Research Ethics Committee.

This cross-sectional study was conducted in 2016. All participants completed a one-time self-reported questionnaire asking about stressful life events, family, and school connection, and depressive symptomology. Eight-graders were targeted in this study because, first, this study aimed to investigate early adolescence, and second, junior high school teachers in Taiwan often observe that children seem to display more stress by eighth grade. Such observations are supported by the school drop-out rate, as statistics show that the highest drop-out rate is often reported during the second year of junior high school (eighth grade; Liu, 1999).

The Han sample in this study all lived in cities, and the Tayal sample all lived in the tribal areas of the Northern Taiwan mountain regions. Han and Tayal participants were the majority in their respective schools.

Table 1 presents the demographic information of the Han and Tayal participants. The family structure, family income, and parental education between the Han and Tayal participants were significantly different. Only 52.7% of the Tayal sample lived with both parents as opposed to 82.5% in the Han sample. More Tayal participants lived in single-parent (31.8%) or grandparent (10.0%) households, as opposed to 15.8 and 0.6%, respectively, for the Han participants, *X*^2^(3)=35.36, *p*<0.001. Family income was measured using the Taiwanese currency (Taiwan New Dollar: TWD) in a six-point scale: below 20,000; 20,001–35,000; 35,001–50,000; 50,001–70,000; 70,001–100,000; and above 100,001. Children this age might still not yet know how much their parent(s) earn; therefore, the number of missing data was high for the family income variable. Despite this, the available data showed that most (69.4%) Tayal household income fell in the lower income range (below 35,000), whereas 63.7% of the Han households were in the higher income range (50,000 and above), *X*^2^(5)=52.90, *p*<0.001. Parental education showed similar distribution as of the family income. Most Tayal parents completed 9- or 12-year education, and significantly, fewer Tayal parents received associate, bachelor’s, or post-graduate degrees [father’s education: *X*^2^(5)=79.01, mother’s education: *X*^2^(5)=76.84, *ps*<0.001].

**Table 1:** Demographic information of the Tayal and Han participants.

	Tayal (*n* =115)	Han (*n* =176)
Age in years (*M*, *SD*)	13.47 (0.52)	13.41 (0.55)
Female (*n*, %)	58 (50.4)	84 (47.7)
Family structure		
Two-parent household (*n*, %)	58 (52.7)	141 (82.5)
Single-parent household (*n*, %)	35 (31.8)	27 (15.8)
Grandparent household (*n*, %)	11 (10.0)	1 (0.6)
Not living with parents or grandparents (*n*, %)	6 (5.5)	2 (1.2)
Family income (Taiwan New Dollar: TWD)		
Below 20,000 (*n*, %)	27 (31.8)	8 (6.5)
20,001–35,000 (*n*, %)	32 (37.6)	16 (12.9)
35,001–50,000 (*n*, %)	15 (17.6)	21 (16.9)
50,001–70,000 (*n*, %)	5 (5.9)	27 (21.8)
70,001–100,000 (*n*, %)	5 (5.9)	22 (17.7)
Above 100,001 (*n*, %)	1 (1.2)	30 (24.2)
Father’s education (years)		
6years (*n*, %)	6 (5.6)	1 (0.6)
9years (*n*, %)	50 (46.3)	23 (13.4)
12years (*n*, %)	44 (40.7)	57 (33.1)
14 to 16years (*n*, %)	6 (5.5)	65 (37.8)
Above 16years (*n*, %)	2 (1.8)	26 (15.1)
Mother’s education (years)		
6years (*n*, %)	10 (9.1)	8 (4.7)
9years (*n*, %)	41 (37.3)	10 (5.9)
12years (*n*, %)	50 (45.5)	65 (38.2)
14 to 16years (*n*, %)	9 (8.2)	72 (42.4)
Above 16years (*n*, %)	0	15 (8.8)

The number of missing data: five Tayal and five Han in family structure, 30 Tayal and 52 Han in family income, seven Tayal and four Han in father’s education, and five Tayal and six Han in mother’s education. Parents’ education: 14–16years is equal to associate or bachelor’s degree level, and above 16years is equal to post-graduate degree level.

### Measures

Measures used in the current study were all adolescent self-reported measures, including depressive symptomology, stressful life events, and connection with family and school. The measure assessing family and school connection was translated into Mandarin by the researcher and back translated into English by professional translation services; no meaningful differences were found.

#### Depressive Symptomology

Depressive symptomology was assessed using the validated translated version of Center for Epidemiologic Studies Depression Scale (CES-D) by Chien and Cheng (1985). The CES-D asks whether, in the past week, the participants had experienced sadness, fear, and other symptoms on a four-point scale (0 = rarely or none of the time, 1 = some or little of the time, 2 = a lot of the time, and 3 = most or all of the time). The sum of the 20 items represents the extent of depressive symptoms experienced. Cronbach’s alpha for depressive symptomatology was 0.88.

#### Stressful Life Events

Stressful life event items were drawn from survey items in the Taiwan Youth Project (TYP). The TYP examined the trajectory of Taiwanese youth development in family, school, and community contexts. Two cohorts (seventh-graders and ninth-graders) were recruited and followed since 2000 (see Yi, 2013 for a description of the TYP). The TYP consisted of experts in the field of adolescent and family research. The stressful life event items were developed based on consensus among experts who had compiled a list of common family and school stressors experienced by Taiwanese adolescents. However, these stressful life event items have not been investigated in research; therefore, their psychometric properties, such as test-reliability, are yet to be confirmed.

Stressful life events were assessed by asking in the past twelve months whether any family events (six items, e.g., parents divorced or separated) or school events (five items, e.g., my grades have been dropping significantly) had happened (0 = none and 1 = yes). Family and school event items were used, respectively, to create both latent profiles and cumulative scores for family and school stress. Latent profiles were created using the latent class analyses, and cumulative scores were created by summing the stressful life event items.

Internal consistencies of the family and school stressful life events were not computed because the six items in the family context and the five items in the school context represent specific events. Although these events might be interlinked (Wagner et al., 1988), we did not expect that all event items would correlate with each other, meaning that the six family-event items, or the five school-event items, are not one-dimensional in structure, and the sum of items variance is likely to be high.

#### Connection With Family and School

Items measuring family connection (six items) and school connection (seven items) were drawn from the Profiles of Student Life-Attitudes and Behaviors Survey (Benson et al., 1998) which were used in the 4-H study of Positive Youth Development (Lerner et al., 2005; Phelps et al., 2009; Bowers et al., 2010). All items were measured on a five-point scale. Example items for family connection are “My parents give me help and support when I need it (1 = strongly disagree, 5 = strongly agree)” and “If you had an important concern about drugs, alcohol, or sex, or some other serious issues, would you talk to your parent(s) about it? (0 = no, 2 = I’m not sure, 4 = yes).” Example items for school connection are “I get a lot of encouragement at my school (1 = strongly disagree, 5 = strongly agree)” and “How often do you feel bored at school? (0 = always, 4 = never).” All items were rescaled to 0–12 following Lerner’s (2010) scoring protocol. Items were then averaged to create the composite score for family connection and school connection, respectively. Cronbach’s alpha for family connection was 0.89 and school connection was 0.85.

### Data Analysis Plan

This study applied person-centered and cumulative approaches to examine stressful life events, and examined the association between stressful experiences, connection, and depressive symptomology. Direct, indirect, and moderating effects of connection on depressive symptomology were examined.

Latent class analyses (LCA) were used to identify the stress profiles experienced at home and in school. Chi-squares and *t*-tests were used to examine racial/ethnic differences in LCA and cumulative stress results, respectively.

A four-way (Family-stress profiles X School-stress profiles X Ethnicity X Gender) on depressive symptomology was conducted first to understand the effects of LCA stress profiles, ethnicity, and gender on depressive symptomology. Gender was included because prior research has suggested that females tend to experience more depressive symptoms than males during adolescence (Kandel and Davies, 1982).

Hierarchical regression analyses were then conducted individually for the Tayal and the Han participants, using either the LCA stress profiles or the cumulative stress scores as variables representing stressful experiences. In the first model, only variables representing stressful experiences were entered. In the second model, connection with family and school was added. In the final model, interaction terms between stressful experience variables and connection variables were entered. The first and second models examined the direct (main) effect of stressful experiences and family and school connection, and the final model further examined the moderating effect of the connection variables.

If any significant moderating effects were found, simple slope analyses were conducted to examine the effect in the high (+1 *SD*) and low (−1 *SD*) connection groups (Aiken and West, 1991).

The indirect effect of connection was tested using the bootstrap method to examine the indirect effect of stressful experiences on depression through connection. Shrout and Bolger (2002) recommend using bootstrap tests to assess mediation (indirect effects) when the sample is small to moderate in size, which was the case in our study. Bootstrap tests use the original dataset to randomly recreate pseudo(bootstrap) samples. Following Shrout and Bolger’s (2002) recommendation, the significance of the bootstrap tests is also reported using 95% confidence intervals instead of the null-hypothesis statistical tests.

## Results

### Latent Class Analyses on Stressful Events Experienced at Home and in School

Latent class analyses were used to identify the stress profiles experienced at home and in school. First, LCA models (Nylund-Gibson and Choi, 2018) were built using the family-event items. A one-class LCA model was first estimated as a comparative baseline for later models. The number (*k*) of classes was increased by one in each of the following models till the model result reached conceptually explanatory and statistically significant solutions. A range of model fit criteria was used to determine the optimal class solution, including Akaike’s information criterion (AIC), Bayesian information criterion (BIC), sample-size-adjusted BIC (SABIC), bootstrapped likelihood ratio test (BLRT), and Vuong-Lo-Mendell-Rubin-adjusted likelihood ratio test (VLMR-LRT). The AIC, BIC, and SABIC are approximate fit indices where lower values indicate better fit. The BLRT and VLMR-LRT are likelihood-based tests, which compare the fit between *k* and *k−1* class solutions. Significant value of *p* for *k* class indicates improving fit from the previous class solution, whereas insignificant value of *p* for *k* class supports the selection of the previous class solution. The same LCA procedure was conducted for the school-event items.

Table 2 shows the fit statistics and classification coefficients for the LCA results for stress experienced at home and in school. It appeared that a 2-class solution was the best fit for stress experienced in school. However, fit indices for stress experienced at home did not converge on a single solution. This is not uncommon in applied settings. Given that information criteria tend to continually decrease with each additional class and the BLRT is shown to be a comparably robust measure (Nylund et al., 2007; Nylund-Gibson and Choi, 2018), the 2-class solution was selected for stress experienced at home.

**Table 2:** Fit statistics and classification coefficients for the LCA results for stress experienced at home and in school.

Number of classes	Family stress			School stress		
	1 class	2 classes	3 classes	1 class	2 classes	3 classes
LL	−702.88	−666.78	−657.95	−563.61	−547.46	−542.81
AIC	1417.77	1359.56	**1355.90**	1137.21	**1116.92**	1119.63
BIC	1439.81	**1407.32**	1429.37	1155.58	**1157.32**	1182.07
SABIC	1420.78	1366.09	**1365.94**	1139.72	**1122.44**	1128.16
BLRT *p*		**<0.001**	0.071		**<0.001**	0.230
VLMR-LRT *p*		**<0.05**	**<0.05**		**<0.001**	0.0.097
Smallest class (%)		21.0%	7.2%		30.9%	12.3%

LL, log-likelihood; AIC, Akaike’s information criterion; BIC, Bayesian information criterion; SABIC, sample-size adjusted BIC; BLRT, bootstrapped likelihood ratio test; VLMR-LRT, Vuong-LO-Mendell-Rubin-adjusted likelihood ratio test; and *p*, *p* value. Bold values indicate best fit for each respective statistic.

Table 3 provides descriptive information of the family-event items between the two LCA profiles. At home, the main difference between the two profiles is that none of the participants in profile 1 reported relationships in conflict between parents or adults at home, whereas 61% in profile 2 had experienced this. Overall, participants in profile 2 seemed to experience more tension at home. The only item that showed no significant differences between the two profiles is “I have family members or friends who have passed away.” For the rest of the family-event items, participants in profile 2 had experienced significantly more of them.

**Table 3:** Descriptive information of stressful life events experienced at home by ethnicity and LCA profiles.

No.	At homes	Ethnicity			LCA profiles					
		Total (*n* =291)	Tayal (*n* =115)	Han (*n* =176)	Profile 1 (63% Han)			Profile 2 (49% Han)		
					Tayal (*n* =85)	Han (*n* =145)	Total (*n* =230)	Tayal (*n* =31)	Han (*n* =30)	Total (*n* =61)
1.	Parents divorced or separated (*n*, %)	27 (9.2%)	**18 (15.9%)**	**9 (5.1%)**	7 (8%)	2 (1%)	9 (4%)	11 (38%)	7 (23%)	18 (31%)
2.	Financial problems (*n*, %)	53 (18.2%)	25 (21.7%)	28 (15.9%)	12 (14%)	12 (8%)	24 (10%)	13 (42%)	16 (53%)	29 (48%)
3.	More arguments/fights among parents/adults at home (*n*, %)	37 (12.7%)	**20 (17.4%)**	**17 (9.7%)**	0 (0%)	0 (0%)	0 (0%)	20 (65%)	17 (57%)	37 (61%)
4.	Parents more frequently absent from home (*n*, %)	30 (10.3%)	13 (11.3%)	17 (9.7%)	3 (4%)	7 (5%)	10 (4%)	10 (32%)	10 (33%)	20 (33%)
5.	More arguments/fights between me and my parents or adults at home (*n*, %)	37 (12.7%)	16 (13.9%)	21 (11.9%)	1 (1%)	6 (4%)	7 (3%)	15 (48%)	15 (50%)	30 (49%)
6.	I have family members or friends who have passed away (*n*, %)	67 (23.0%)	**39 (33.9%)**	**28 (15.9%)**	28 (33%)	20 (14%)	48 (21%)	11 (35%)	8 (27%)	19 (31%)
Average cumulative stress score at home (*M*, *SD*)		0.84 (1.11)	**1.12 (1.18)**	**0.67 (1.03)**	0.61 (0.71)	0.30 (0.52)	0.41 (0.61)	2.59 (1.02)	2.43 (1.04)	2.51 (1.02)

Bold values indicate significant racial/ethnic differences according to Chi-square or *t*-test results. Percentage is calculated based on the number of total, Tayal, and Han participants in the respective columns.

Table 4 provides descriptive information of the school-event items between the two LCA profiles. In school, the main difference between the two profiles is that the source of stress for participants in profile 1 was from school performance solely. Participants in profile 2, however, had experienced not only performance stress (i.e., my grades have been dropping significantly) but also other types of stress, such as relational stress (i.e., I fell out with my good friends; I was bullied in class) and opportunity restriction (i.e., I was not allowed to be part of school teams or participate in activities at school).

**Table 4:** Descriptive information of stressful life events experienced in school by ethnicity and LCA profiles.

No.	In schools	Ethnicity			LCA profiles					
		Total (*n* =291)	Tayal (*n* =115)	Han (*n* =176)	Profile 1 (61.2% Han)			Profile 2 (59.9% Han)		
					Tayal (*n* =78)	Han (*n* =123)	Total (*n* =201)	Tayal (*n* =36)	Han (*n* =54)	Total (*n* =90)
1.	I fell out with my good friends (*n*, %)	62 FLCA (21.3%)	23 (20.0%)	39 (22.2%)	0 (0%)	0 (0%)	0 (0%)	23 (64%)	39 (72%)	62 (69%)
2.	My grades have been dropping significantly (*n*, %)	112 (38.5%)	48 (41.7%)	64 (36.6%)	28 (36%)	41 (33%)	69 (34%)	20 (55%)	23 (43%)	43 (48%)
3.	I was bullied in class (*n*, %)	26 (8.9%)	10 (8.8%)	16 (9.1%)	0 (0%)	0 (0%)	0 (0%)	10 (28%)	16 (30%)	26 (29%)
4.	I changed school (*n*, %)	15 (5.2%)	6 (5.2%)	9 (5.1%)	0 (0%)	0 (0%)	0 (0%)	6 (17%)	9 (17%)	15 (17%)
5.	I was not allowed to be part of school teams or participate in activities at school (*n*, %)	20 (6.9%)	**13 (11.3%)**	**7 (4.0%)**	0 (0%)	0 (0%)	0 (0%)	13 (36%)	7 (13%)	20 (22%)
	Average cumulative stress score in school (*M*, *SD*)	0.80 (0.90)	0.86 (0.94)	0.77 (0.88)	0.36 (0.48)	0.33 (0.47)	0.34 (0.48)	1.94 (0.75)	1.79 (0.75)	1.85 (0.75)

Bold values indicate significant racial/ethnic differences according to Chi-square or *t*-test results. Percentage is calculated based on the number of total, Tayal, and Han participants in the respective columns.

### Racial/Ethnic Differences in Stressful Experiences

Tables 3 and 4 provide descriptive information of the items and cumulative stress scores (sum of the number of items) at home and in school by ethnicity and LCA profiles.

Chi-square and *t*-tests were used to examine the relation between ethnicity and stressful experiences (i.e., family- and school-event items, family and school cumulative stress scores, and family and school stress LCA profiles). The results showed that the Tayal sample experienced significantly more parental divorce [*X*^2^(1)=9.40, *p*<0.01], arguments or fights between parents [*X*^2^(1)=3.75, *p*<0.06], and deaths of family members or friends [*X*^2^(1)=7.92, *p*<0.05]. They also experienced more opportunity restriction, not being able to participate in school teams or other school activities [*X*^2^(1)=5.83, *p*<0.05]. With respect to the family-stress LCA profiles, 27% of Tayal participants as opposed to 17% Han participants were classified into profile 2 [*X*^2^(1)=4.12, *p*<0.05]. The Tayal participants appeared to experience more cumulative stressful events at home [*t* (285)=3.41, *p*<0.01]. But, in school, there were no significant racial/ethnic differences in the cumulative number of stressful events or between the school-stress LCA profiles.

### Analysis of Variances Examining the Association Between Depressive Symptomology, Stress Profiles, Ethnicity, and Gender

A four-way ANOVA (Family-stress profiles×School-stress profiles×Ethnicity×Gender) was conducted on depressive symptomology. The results showed significant effects of family-stress LCA profile [*F*(1, 263)=12.17, *p*<0.01] and school-stress LCA profile [*F*(1, 263)=11.64, *p*<0.01], as well as a significant interaction effect of gender and family-stress LCA profile [*F*(1, 263)=5.23, *p*<0.05]. Participants in profile 2 (*M*_family_=20.76, *SD*_family_=10.47; *M*_school_=19.92; *SD*_school_=10.54) had significantly higher depression scores than those in profile 1 (*M*_family_=13.99, *SD*_family_=9.09; *M*_school_=13.55; *SD*_school_=8.82). Female participants (*M*_female×family profile 2_=23.13, *SD*_female×family profile 2_=11.60) classified in family stress LCA profile 2 had higher depression scores than the male participants (*M*_male×family profile 2_=17.14, *SD*_male×family profile 2_=7.30). Ethnicity did not show any significant effect on depression.

### Hierarchical Regression Analyses Examining the Main, Indirect, and Moderating Effects of Connection

Table 5 shows descriptive information of and bivariate correlations between depressive symptomology, stressful experience, and connection variables, respectively, for the Tayal and Han participants. Hierarchical regression analyses were conducted individually for the Tayal and Han samples, having depressive symptomology regressed on the stressful experience variables (model 1; LCA stress profiles and cumulative stress scores were examined in separate models), family and school connection variables (model 2), and interaction terms between stressful experience and connection variables (model 3). Continuous variables (i.e., cumulative stress scores and connection variables) were mean centered before creating the interaction variables.

**Table 5:** Correlations and descriptive information among depression, stress, and connection variables^a^.

No.		1	2	3	4	5	6	7
1.	Depression	–	0.26^**^	0.29^**^	0.23^**^	0.35^***^	−0.36^***^	−0.31^***^
2.	Family stress LCA profile 2	0.32^**^	–	0.20^**^	0.71^***^	0.24^**^	−0.15^*^	−0.01
3.	School stress LCA profile 2	0.30^**^	0.21^*^	–	0.20^**^	0.75^***^	−0.07	−0.19^*^
4.	Cumulative family stress score	0.23^*^	0.70^***^	0.13	–	0.27^**^	−0.18^*^	0.04
5.	Cumulative school stress score	0.28^**^	0.19^*^	0.78^***^	0.11	–	−0.15^*^	−0.15^*^
6.	Family connection	−0.46^***^	−0.40^***^	−0.26^**^	−0.33^***^	−0.22^*^	–	0.45^***^
7.	School connection	−0.36^***^	−0.15	−0.34^***^	−0.12	−0.31^**^	0.53^***^	–
Tayal (*M, SD* or *n*, %)		15.32 (9.78)	**31 (27%)**	37 (32.2%)	**1.12 (1.18)**	0.86 (0.94)	8.24 (2.85)	**8.25 (2.03)**
Han (*M, SD* or *n*, %)		15.56 (9.82)	**30 (16.7%)**	53 (29.4%)	**0.67 (1.03)**	0.77 (0.88)	8.35 (2.46)	**7.68 (2.10)**

Han results are reported above the diagonal; Tayal results are reported below the diagonal. Bold values indicate significant racial/ethnic differences according to Chi-square or *t*-test results.

* *p*<0.05;

** *p*<0.01;

*** *p*<0.001.

a Spearman rho was used to report correlation results.

Tables 6 and 7 show the results of the hierarchical regression analyses. In model 1, the family and school stress LCA profiles (Table 6) and the cumulative school stress scores (Table 7) were significantly associated with depressive symptomology for both the Tayal (*β*_family stress profile_=0.24, *β*_school stress profile_=0.22*_,_ β*_cumulative school events_ *=0*.24; *ps*<0.05) and Han participants (*β*_family stress profile_=0.23, *β*_school stress profile_=0.27*, β*_cumulative school events_ *=0*.29; *ps*<0.01), but the cumulative family stress score was significant only for the Han sample (*β*_cumulative family events_ *=0*.17; *p*<0.05).

**Table 6:** Hierarchical regression results on Tayal’s and Han’s depression using family and school stress LCA profiles.

			Family stress profile 2	School stress profile 2	Family connection	School connection	Family stress profile 2×Family connection	Family stress profile 2×School connection	School stress profile 2×Family connection	School stress profile 2×School connection	
Tayal	Model 1	*B*	5.26	4.57							*F* (2, 107)=8.11^***^ *Adjusted R*^2^=0.12
		*SE*	2.04	1.99							
		*β*	0.24^*^	0.22^*^							
	Model 2	*B*	2.55	2.66	−0.89	−0.75					*F* (4, 105)=8.27^***^ *Adjusted R*^2^=0.21
		*SE*	2.12	1.96	0.37	0.49					
		*β*	0.12	0.13	−0.26^*^	−0.16					
	Model 3	*B*	3.24	2.39	−1.27	−0.109	0.34	0.38	1.17	−2.24	*F* (8, 101)=5.24^***^ *Adjusted R*^2^=0.24
		*SE*	2.31	2.09	0.53	0.68	0.79	0.75	1.08	0.97	
		*β*	0.15	0.11	−0.38^*^	−0.02	0.07	0.08	0.12	−0.29^*^	
Han	Model 1	*B*	6.01	5.80							*F* (2, 166)=14.17^***^ *Adjusted R*^2^=0.14
		*SE*	1.92	1.58							
		*β*	0.23^**^	0.27^***^							
	Model 2	*B*	5.19	5.55	−1.15	−0.31					*F* (4, 164)=13.64^***^ *Adjusted R*^2^=0.23
		*SE*	1.84	1.52	0.31	0.37					
		*β*	0.20^**^	0.26^***^	−0.29^***^	−0.07					
	Model 3	*B*	4.93	5.56	−1.19	−0.39	−0.38	0.56	0.35		*F* (7, 161)=7.83^***^ *Adjusted R*^2^=0.22
		*SE*	1.89	1.55	0.36	0.41	0.81	0.68	0.96		
		*β*	0.19^*^	0.26^**^	−0.30^**^	−0.08	−0.04	0.06	0.03		

Connection variables are mean centered. Due to high correlations between school connection and the interaction term (School stress profile 2×School connection) in the Han sample, “School stress profile 2×School connection” was dropped to avoid multi-collinearity problems.

* *p*<0.05;

** *p*<0.01;

*** *p*<0.001.

**Table 7:** Hierarchical regression results on Tayal’s and Han’s depression using cumulative family and school stress scores.

			Family stress score	School stress score	Family connection	School connection	Family stress score×Family connection	Family stress score×School connection	School stress score×Family connection	School stress score×School connection	
Tayal	Model 1	*B*	1.13	2.62							*F* (2, 104)=4.70^*^ *Adjusted R*^2^=0.07
		*SE*	0.79	1.03							
		*β*	0.14	0.24^*^							
	Model 2	*B*	0.15	1.81	−1.09	−0.68					*F* (4, 102)=7.39^***^ *Adjusted R*^2^=0.19
		*SE*	0.77	0.99	0.37	0.49					
		*β*	0.02	0.17	−0.31^**^	−0.14					
	Model 3	*B*	0.80	1.77	−1.19	−0.62	0.43	0.46	−0.29	−1.21	*F* (8, 98)=4.56^***^ *Adjusted R*^2^=0.21
		*SE*	0.85	1.01	0.40	0.51	0.32	0.49	0.47	0.62	
		*β*	0.10	0.16	−0.34^**^	−0.13	0.15	0.10	−0.07	−0.20^ǂ^	
Han	Model 1	*B*	1.69	3.36							*F* (2, 163)=13.84^***^ *Adjusted R*^2^=0.14
		*SE*	0.74	0.88							
		*β*	0.17^*^	0.29^***^							
	Model 2	*B*	1.55	2.90	−1.00	−0.47					*F* (4, 161)=12.77^***^ *Adjusted R*^2^=0.22
		*SE*	0.73	0.85	0.31	0.37					
		*β*	0.16^*^	0.25^**^	−0.26^**^	−0.10					
	Model 3	*B*	1.33	3.60	−0.87	−0.52	−0.24	0.35	−0.29	0.64	*F* (8, 157)=7.07^***^ *Adjusted R*^2^=0.23
		*SE*	0.76	0.90	0.32	0.38	0.31	0.40	0.41	0.46	
		*β*	0.14	0.31^***^	−0.22^**^	−0.11	−0.06	0.07	−0.06	0.11	

Cumulative stress score and connection variables are mean centered.

ǂ *p*=0.056;

* *p*<0.05;

** *p*<0.01;

*** *p*<0.001.

#### Tayal Regression Results

**Figure 1:**
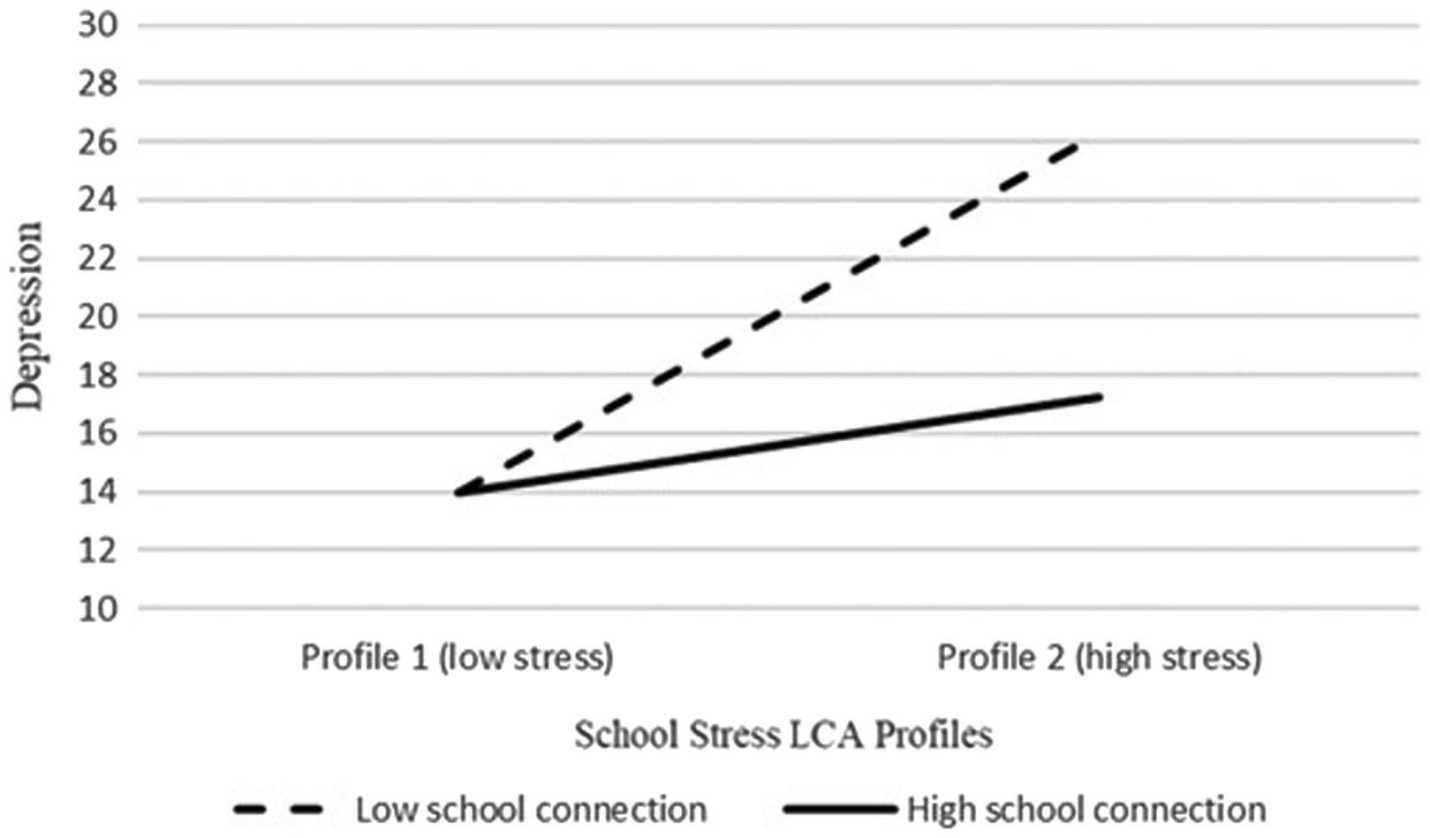
The relation between school stress LCA profiles and depression scores across the high (+1 *SD*) and low (–1 *SD*) school connection groups in the Tayal sample.

For the Tayal sample, after factoring in the effect of connection and the interaction effects, the family and school stress LCA profiles (see the Tayal part of models 2 and 3 in Table 6) and the cumulative school stress scores (see the Tayal part of models 2 and 3 in Table 7) were no longer significant. Rather, a direct effect of family connection and a significant to marginal moderating effect of school connection on school stress were found. Simple slope analyses were conducted examining the significant moderating effect of school connection on the relation between school stress LCA profiles and depression. The results showed that the depression score decreased more in the high school connection (+1 *SD*) group (*B*=−21.42, *SD*=10.55, *p*=0.042) than the low school connection (−1 *SD*) group (*B*=−12.35, *SD*=6.28, *p*=0.049). Figure 1 shows the relationship between the school stress LCA profiles and depression in the Tayal sample, indicating that Tayal participants in the high school stress profile (profile 2) reported lower depression scores when they were in the high school connection group as opposed to their counterparts in the low school connection group.

**Figure 2:**
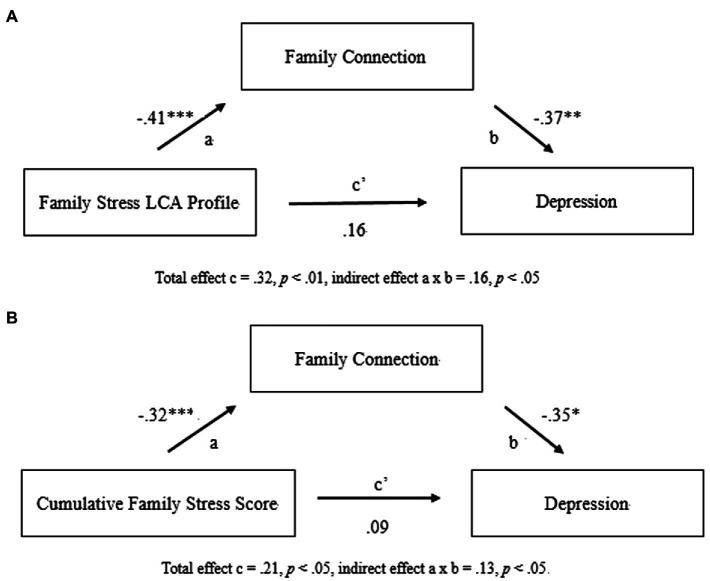
Abbreviated models showing the indirect relationship between family stress, family connection, and depression variables in the Tayal sample (Panel **A** shows family stress LCA profile, and Panel **B** shows cumulative family stress score). Regression coefficients are reported using standardized value. The regression coefficients here might be slightly different from the values reported in Tables 6 and 7, because the values here were obtained using bootstrap tests. ^*^p <0.01, ^**^p <0.01, and ^***^p <0.001.

Bootstrap tests were used to examine the indirect effect of connection. Significant indirect effects of family connection were found at < 0.05 significance level explaining the relationship between the family stress LCA profiles and depression (95% CI [0.03, 0.27]) and the relationship between the cumulative family stress scores and depression (95% CI [0.002, 0.22]) in the Tayal sample. Figure 2 shows a pictorial illustration of the bootstrap results on the indirect effect of family connection.

#### Han Regression Results

For the Han sample, after factoring in the effect of connection (see the Han part of models 2 and 3 in Tables 6 and 7), a direct effect of family connection was found, but the family and school stress LCA profiles and the cumulative school stress scores remained significantly associated with depressive symptomology, and no indirect or moderating effects of connection were found.

## Discussion

As the reporting rate of depressive affect and symptoms increases substantially in early adolescence, this study, by examining stressful life events experienced by 13- to 14-year-old youths at home and in school, aimed to draw more understanding of the association between stressful experiences and depressive symptomology during early adolescence. Using cumulative and person-centered approaches, this study examined stressful experiences at home and in school. Connection experienced in both family and school contexts was also examined, as PYD (e.g., Phelps et al., 2007; Holsen et al., 2017) and adolescent stress research (e.g., Grant et al., 2006) have both identified that connection or social support might decrease depressive symptoms. Furthermore, the investigation of the stressor-depression association was conducted in a racial/ethnic context. In addition to sampling youth from the majority Han population in Taiwan, youth from the indigenous Tayal tribe was part of this study, providing more racial/ethnic understanding of the association between stressful experiences, connection, and depressive symptoms.

Overall, the findings from this study confirm the association between stressful experiences and depressive symptomology. Results using both the cumulative and person-centered stress variables show that participants who had experienced more number of stressful events or were classified into the high stress profiles (profile 2) at home or in school appear to show significantly more depressive symptoms. Our results are in line with Wagner et al. (1988) and Seiffge-Krenke (2000), supporting the use of an integrative model when investigating stressful experiences.

However, while cumulative and person-centered approaches both proved to be useful, our results suggest that the distinct stress profile identified by a person-centered approach might be more meaningful than a unidimensional cumulative approach. Using LCAs, this study uncovered distinct patterns of stressors within a given group and thus provided more pattern-based contextual understanding of stressors experienced by early adolescents at home and in school. For example, according to the LCA results on school stresses, it appears that “my grades have been dropping” might be commonly experienced by all junior high school students in Taiwan, as 34 and 48%, respectively, in the low and high school-stress profiles experienced this. A cumulative approach is limited because it provides understanding only in terms of the number of events. Although the overall results of this study did not differ much between the two approaches, this study recommends including person-centered pattern-based methods in the future research.

Our results also show that family connection significantly lowered depressive symptomology, regardless of ethnicity. As with Grant et al. (2006) and Tsai et al. (2018), this study too found that positive family relationships are imperative to children’s psychological adjustment. School connection, on the other hand, did not demonstrate any significant direct effect on depression, again reinforcing the important role of family relationships when coping with adolescent depression.

However, the differential findings between the Tayal’s and Han’s results on the indirect and moderating effects of connection revealed a greater complexity of the role of connection.

The Tayal results show that family stresses seem to lead to depression through family connection, as in Figures 2A,B, only significant indirect effects (path a×b) were found, and direct effects (path c’) of family stresses were not significant. This finding suggests that Tayal family relationships might be particularly vulnerable to critical family, marital, or financial problems and daily family frictions. Indeed, significantly more Tayal participants were classified into the high family stress profile, and significantly more reported a greater number of stressful events at home than their Han counterparts. Our results show that Tayal participants experienced more domestic frictions, parental divorce, and deaths, all of which were likely to create strains on family relationships, consequently increasing the occurrence of depressive symptoms in Tayal youth. Other mediator research investigating the mediating (indirect) role of interpersonal relationships has reported similar findings, showing that stressful experiences affected psychological functioning through the disruption of important family relationships (Grant et al., 2006).

Moderating effects were observed only in the Tayal sample, showing that school connection moderated the association between Tayal participants’ experiences of school stresses and depression. Connection with teachers and peers in school appears to buffer the level of stress experienced by Tayal adolescents in school. Those classified in the high school stress profile appear to have lower depression when having high school connection as opposed to those having low school connection.

In our Han sample, no indirect and moderating effects of family or school connection were found. This is surprising, and we wonder whether there might be other factors through which stress affected depression in the Han sample. Some mediator research has found evidence of variables, such as perceived competence, self-esteem, problem solving skills, or negative cognitions, suggesting that stressful experiences might lead to depression through variables representing the child’s perceived self and competence or through other characteristics of the child (Cheng and Lam, 1997; Tram and Cole, 2000; Chang and Sanna, 2003). Future research might include variables measuring not only the child’s relationships with others, but also the child’s characteristics in their investigation of Han children, so that more can be understood about which personal, relational, or contextual factors might mediate or moderate the effects of stress on Han children’s depressive symptomology.

Similar to Motti-Stefanidi (2015), this study found that ethnicity alone might not predict psychological adjustment, such as depressive symptomology. We were somewhat surprised to find that, considering the scope of family stressors experienced by the Tayal participants, they did not report more depressive symptoms than the Han participants. We wonder whether there might be other factors supporting the Tayal children which this study did not measure. One possible factor is tribal/community connection. Yellow Bird (2013) has suggested that the nature of hunter-gatherer’s lives depended strongly on collectivism and communal sharing. The Tayal sample in this study lived in their tribal villages, surrounded mainly by their fellow Tayal people. It is likely that the connection with their fellow tribal youth and tribal members has made them resilient to the adverse effects of stressful family lives. Future research on indigenous populations might consider including tribal/community connection in their investigation of stressful experiences and depressive symptomology.

Lastly, although this study did not particularly focus on gender differences, the results show that girls who were classified into the high family stress profile experienced more depressive symptoms. The home atmosphere of participants classified in the high family stress profile (profile 2) might be more unstable, with constant quarrels or fights between family members and between the participants and other family members. Females, particularly adolescent girls, tend to be more sensitive to interpersonal conflicts (Liu and Alloy, 2010). Our results thus suggest that adolescent girls who are constantly in situations of interpersonal discord or animosity may be particularly vulnerable to depression.

## Conclusion

This study examined the association between stressful experiences, connection, and depressive symptoms during early adolescence across samples of Han and indigenous (Tayal) youth. Using integrative models of stress, we found that stressful experiences are clearly linked to depressive symptomology in early adolescence. Further, family connection was important to both Tayal and Han youth supporting them to cope with depressive symptoms. However, Tayal youth might be particularly vulnerable to family stressful events because divorce, death, financial strain, or family tension likely disrupt their connection with their parents and thereby minimize the protective function of family relationships. The Tayal sample in this study had significantly lower socio-economic status than the Han sample. Disadvantaged status is associated with multiple stressors (Smith and Carlson, 1997; Meyer et al., 2008). This was evident in our Tayal participants’ reports of stressful family events, which put Tayal youth in greater risk of depression.

Overall, this study supports findings from prior research (e.g., Seiffge-Krenke, 2000; Grant et al., 2006; Tsai et al., 2018) showing that stress adversely affects adolescent psychological outcomes and that family connection and relationships are imperative to adolescent mental health. During early adolescence, it is important for parents and family members to be aware of the stresses their children experience in their daily lives; therefore, timely support could be provided to avoid the onset of adolescent depression.

Our findings have important mental health policy implications for adolescents from different racial/ethnic backgrounds. It appears that stress might affect mental health through specific pathways for young people from different racial/ethnic backgrounds. Contextual understanding of the socio-historical, familial, educational, and economic aspects of lives in which specific young people live is important, so that mental health interventions can be tailored specifically for young people in specific racial, social, and economic contexts. For example, the Tayal participants in our study might benefit from mental health policies which aim to increase school connection and lower family financial difficulties.

This study has its limitations, however. Firstly, the sample size of this study is fairly small, which constrains the generalizability of our results to the broader indigenous and Han youth in Taiwan. Secondly, the stressful experiences measured in this study did not ask about perceived discrimination, or conflicts or constant shifting between the indigenous and mainstream cultures, which have been identified by other researchers (Whitbeck et al., 2002; Mossakowski, 2003; LaFromboise et al., 2006) as common stressful experiences for indigenous young people. Thirdly, this study included only participants from the Tayal tribe who were residing with their fellow tribal members, so caution is advised when using these results with other indigenous tribes, or applying them to indigenous people who have relocated to majority Han urban areas. Fourthly, the sample size did not allow us to test for measurement invariance between the Han’s and Tayal’s ratings across the instruments used in this study. Measurement invariance helps confirm whether the two samples interpret the measured items in a conceptually similar manner. Every child in Taiwan receives a standard curriculum of education in Mandarin; therefore, both our Han and Tayal samples are fluent in written and oral Mandarin, which is the language used in the questionnaire. Additionally, because the description of items used in this study (stressful experiences, connection, and depressive symptomology) is straightforward common terms used in daily life, it is likely that the Han and Tayal participants had a similar conceptual understanding of them too. Therefore, despite the fact that measurement invariance was not tested in this study due to limit in sample size, it is possible that there were no marked differences in participants’ understanding of the questionnaire items. However, we would still recommend that the future research addresses this and conducts measurement invariance tests between the Taiwanese Han and indigenous populations where possible. Fifthly, this study applied a cross-sectional design, making it impossible to infer the causal link between variables. Future research should aim to study stress, connection, and depression longitudinally, so the temporal order of the factors can be determined. Lastly, this study examined only the way connection might mitigate depressive symptoms. Adolescent research has identified other factors which might potentially mitigate symptoms of depressive symptomology, such as negative cognitions (Chang and Sanna, 2003), ethnic identity or cultural orientation (Neblett et al., 2012; Cavanaugh et al., 2019), or prosocial behavior (Davis et al., 2016), and we encourage the inclusion of these factors in the future research.

Research on indigenous children and youth is scarce; therefore, in spite of these limitations, this study contributes to current research and future discussions of the relationship between stressful experiences, connection, and depressive symptomology on young people from diverse backgrounds and cultures.

## Data Availability Statement

The raw data supporting the conclusions of this article will be made available by the authors, without undue reservation.

## Ethics Statement

The studies involving human participants were reviewed and approved by the National Taiwan University Research Ethics Committee. Written informed consent to participate in this study was provided by the participants’ legal guardian/next of kin.

## Author Contributions

P-JY designed and carried out the study and statistical analyses, and drafted, edited, and revised the manuscript.

## Conflict of Interest

The author declares that the research was conducted in the absence of any commercial or financial relationships that could be construed as a potential conflict of interest.

## Publisher’s Note

All claims expressed in this article are solely those of the authors and do not necessarily represent those of their affiliated organizations, or those of the publisher, the editors and the reviewers. Any product that may be evaluated in this article, or claim that may be made by its manufacturer, is not guaranteed or endorsed by the publisher.
